# Establishing evidence-based pharmacologic treatments for neonatal abstinence syndrome: A retrospective case study

**DOI:** 10.1017/cts.2022.431

**Published:** 2022-07-25

**Authors:** Sarah K. Brewer, Jonathan M. Davis, Rachana Singh, Lisa C. Welch

**Affiliations:** 1 Tufts Clinical and Translational Science Institute, Tufts University, Boston, MA, USA; 2 Tufts Medical Center, Boston, MA, USA

**Keywords:** Retrospective case study, successful translation, CTSA, neonatal abstinence syndrome, pediatric pharmacology

## Abstract

Translation of research discoveries into health impact can take many years, creating delays in improving clinical outcomes. One approach to promoting timely translation is to examine successful cases in order to understand facilitators and strategies for overcoming barriers. We examined the development of evidence-based management for neonatal abstinence syndrome (NAS) at one academic medical center, with a primary focus on pharmacologic treatment. Despite a substantial increase in NAS case incidence starting in the early 2000s, significant sociocultural, policy, and regulatory barriers limited collaborative NAS research. Facilitators for translation encompassed: 1) pursuing research of societal interest, 2) building an effective interdisciplinary team, 3) intentionally linking clinical, research, and advocacy efforts, 4) broad stakeholder engagement across clinical, policy, and research arenas, and 5) leveraging local resources. Challenges included lack of commercially available U.S. Food and Drug Administration approved neonatal drug formulations, legal and regulatory barriers related to off-label and illicit use of opioids, recruitment for a treatment associated with drug withdrawal syndromes, misalignment of research design needs with real-world scenarios, and episodic funding. Benefits of successful translation included improvements in clinical care, reduced healthcare costs related to NAS, and enhanced legislative, policy, and research strategies to support broader neonatal investigations.

## Introduction

The translation of research discoveries into improvements in clinical care and public health takes approximately 17 years [1]. Delays in translation of therapeutic options lead to suboptimal care, poor health outcomes among patients, and societal harm in the form of inefficient use of resources. One approach to support efficient translation is to examine cases of translational success in order to identify the underlying factors that contribute to progress as well as barriers that can be minimized for future researchers. This study highlights the case of one research team identifying evidence-based management options for pharmacologic treatment of neonatal abstinence syndrome (NAS).

Amidst the opioid epidemic and an increasing incidence of NAS in Massachusetts, researchers at Tufts Medical Center in 2009 began to advance NAS research on two distinct interacting levels. Locally, the team built a research agenda starting with a study to understand disparate outcomes in neonates with NAS [2] and expanding it to lead the first multicenter comparison trial testing methadone versus morphine to treat NAS [3]. Since none of the drugs used to treat NAS are approved by the U.S. Food and Drug Administration (FDA) for use with infants, the team encountered significant barriers, described below. These barriers reinforced the need for structural changes to enable feasibility of research with neonates in general but more specifically for NAS. In recent years, the team has catalyzed changes in the wider landscape of clinical research in neonates nationally and internationally. Although this case focuses on NAS research, it elucidates key factors and strategies to achieving translational success that may be adaptable for other pediatric diseases.

## Health Problem and Relevance of the Intervention

### Effects of NAS

NAS occurs due to physiologic dependence of neonates after prolonged *in-utero* exposure to opioids and other psychotropic drugs. Signs of NAS include irritability, sweating, poor feeding, low birthweight, growth failure, seizures, tremors, and respiratory distress which result in prolonged hospitalization and poor parent-infant bonding [4]. Clinicians expect negative short- and long-term neurodevelopmental impact from NAS, though additional research needs to be done [4,5]. In addition to human costs, NAS carries substantial economic costs. In 2016, U.S. hospital charges for NAS totaled $2.5 billion, with 83.8% of neonates with NAS covered by Medicaid [6].

### Historical and Social Context of NAS and Clinical Approaches

**Fig. 1.**
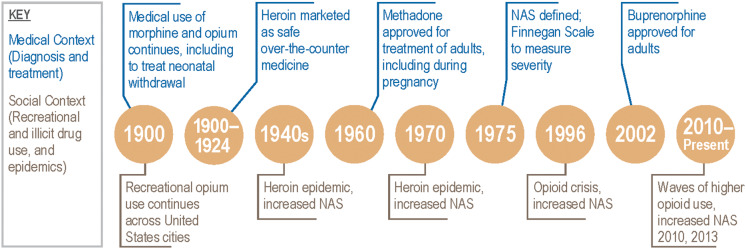
Intersecting historical trajectories for evidence-based (pharmacological) treatment for neonatal abstinence syndrome (NAS).

In the context of recreational, illicit, and medical use of opioids, signs of NAS have been reported for over a century (Fig. 1). The earliest cases of “congenital morphinism” noted in the medical literature were among neonates born to mothers using opium and heroin. By the early 1900s, opium and morphine were used as treatment for NAS [7,8]. During this time period, heroin was marketed as a safe over-the-counter medicine, including for use by pregnant people.[7]

The heroin epidemic beginning in the 1940s and lasting throughout the 1970s brought increased medical attention to NAS [8]. In 1975, NAS was formally defined in the medical literature and one of the first assessment tools, the Finnegan Neonatal Abstinence Scoring System (FNASS), was established to measure severity [9]. Beginning in the 1960s, medication-assisted treatment (MAT) in the form of methadone was prescribed to adults with opioid use disorder (OUD), including during pregnancy. In 2002, another long-acting opioid, buprenorphine, was approved for MAT in adults [10]. While significant improvements in pregnancy-related outcomes occurred with MAT, neonates exposed to methadone and buprenorphine *in utero* were still at-risk for developing NAS [11]. There continued to be a lack of evidence-based standardized protocols for monitoring and treating these high-risk mothers and their neonates.

Since the late 1990s, increases in opioid, heroin, and fentanyl use have occurred, with higher increases among Caucasians than among Blacks or Hispanics [12,13]. Not surprisingly, increases in use contributed to a significant rise in OUD among pregnant people and NAS in neonates [13]. Rates of NAS among hospital-born neonates increased from 1.6 cases per 1,000 births in 2004 to 8.8 cases per 1,000 births in 2016 [14]. By 2016, a neonate with NAS was born every fifteen minutes in the United States [14].

With limited data, some clinicians were using MAT medications (which were formulated and approved for adults with OUD) to treat NAS. Doses were estimated based on the neonate’s FNASS scores, birth weight, or a combination of both and adjusted as needed to manage relevant signs and slowly wean neonates off of the medication [5]. From 2012 to 2013, 87% of hospitalized neonates with NAS received pharmacologic treatment, with 72% receiving morphine, 15% receiving methadone, and less than 1% receiving buprenorphine [15]. Yet, the safety and efficacy of these treatments for neonates were unknown. Although a 2014 analysis of administrative and clinical records suggested that methadone treatment was associated with shorter length of treatment and shorter hospital stay [16], the lack of rigorous clinical trials with an appropriate neonatal formulation led to nonstandardized, nonevidence-based treatment protocols.

The gap in rigorous clinical trials to inform a standard treatment protocol for NAS persisted for decades, possibly due to two reasons. First, the pervasive cultural stigma surrounding drug use made it difficult to access research infrastructure and funding to study prenatal opioid use. Second, even for non-stigmatized health conditions affecting neonates, research infrastructure was lacking, including funding, regulatory approvals, and policies supporting research in this vulnerable population.

### Intervention in Context

The dramatic increase in cases over the last three decades fueled a need for an evidence base for clinical management of NAS including 1) best practices for non-pharmacologic and pharmacologic treatments; 2) safe commercially available neonatal formulations of pharmaceutical agents; 3) an objective, standardized tool to measure NAS severity; and 4) knowledge about neurodevelopmental outcomes [4,17].

A team at Tufts Medical Center formulated a multipronged approach to contribute to evidence-based protocols for treating NAS. While this case focuses primarily on the team’s progress towards a widely available pharmacologic intervention for neonates, their parallel work on non-pharmacologic treatments established their expertise in the area. In addition, the team’s advocacy efforts helped to develop policies and build a culture to support neonatal research, which was essential to facilitating translational progress.

## Case Study Methods

Methods to develop the case included a literature review and key informant interviews. Initially, two analysts (LCW, SKB) reviewed the NAS literature to develop an understanding of the case and its timeline and then conducted semi-structured interviews with research team members at the academic medical center. This included the lead neonatologist and investigator (JMD), a clinician-investigator conducting research on non-pharmacologic and pharmacologic treatments for NAS (RS), and a senior statistician affiliated with the local Clinical and Translational Science Institute (CTSI). Interview recordings were transcribed, coded, and analyzed using a consensus-based approach. Findings were summarized following the process for retrospective case study analyses as described by Dodson et al., and impacts of the case were categorized according to the Translational Science Benefits Model (TSBM) [18,19]. Following the TSBM approach, demonstrated impacts (i.e., “those that have been observed and are verifiable”) and potential impacts (i.e., “those logically expected with moderate to high confidence”) were included [19]. Finally, online searches of third-party information related to funding awards and ongoing clinical trials assisted in completing the timeline. The study was approved by the Case Western Reserve University Institutional Review Board

## Key Events

**Fig. 2.**
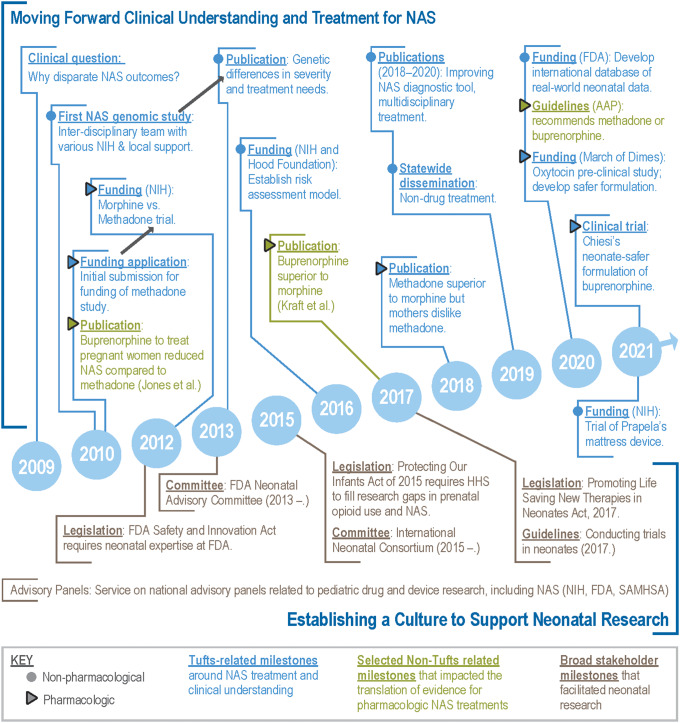
Key milestones toward an evidence-based (pharmacologic) treatment for neonatal abstinence syndrome (NAS).

During the rise of NAS cases in the early 2000s, a clinical question sparked the research team’s multifaceted search for evidence-based treatments (Fig. 2; see Supplementary Material for black-and-white version). A neonatal-perinatal medicine fellow observed that some neonates with NAS had better outcomes than others, but the reason behind these disparate outcomes was unknown. An interdisciplinary team was formed to investigate genomic associations, relying on various sources of funding from the investigators’ institution and the National Institutes of Health (NIH). The results demonstrated potential genetic differences in severity and treatment needs for NAS and the publication was promoted by the Journal of the American Medical Association in a media briefing [2,20]. After initiating the genomics study, the team began designing the first multisite, randomized, double-blind, controlled trial comparing morphine and methadone to treat NAS. The team’s evolving expertise in NAS clinical care and research created opportunities to advocate on behalf of the mother–infant dyad within the U.S. policy arena.

The team initially focused on the lack of approved medications for NAS during a discussion with representatives at the FDA. This advocacy helped in passing the FDA Safety and Innovation Act (FDASIA) of 2012, which required the FDA Office of Pediatric Therapeutics to include experts in pediatric epidemiology and neonatology. This legislation supported the expansion of research with neonates by ensuring those best equipped to evaluate the benefits and risks of this research would be involved in decision-making. The Act also made permanent incentives for the development of pediatric pharmaceutical treatments.

In 2012, the research team received NIH funding to conduct the first clinical trial to compare the safety and efficacy of methadone versus morphine to treat NAS. The multicenter study enrolled 116 infants at eight U.S. hospitals [3]. To enhance safety, the study used a commercial preservative-free formulation of morphine and a study-specific alcohol-free formulation of methadone, which received Investigational New Drug approval from the FDA. As this trial was underway, the team expanded its contributions by supporting non-pharmacologic NAS treatments. From 2014 to 2018, the team contributed to testing a simplified 8-item version of the Finnegan Scale [21] and in 2016 received funding to establish risk assessment models to predict NAS severity and guide precision treatment decisions.

Between 2013 and 2017, the team continued building on their established expertise in clinical care and research to advocate for a culture of research on improving neonatal outcomes, including NAS research. Following the passage of the FDASIA, in 2013 a team member accepted a leadership role and established the Neonatal Advisory Committee at the FDA. This led to a collaboration with a first-term Congressional Representative who sought to de-stigmatize and support mothers with OUD so they could seek treatment and receive prenatal care without legal consequences. A research team member helped to draft the Protecting Our Infants Act of 2015 [22] and to garner Congressional support, testified about the need for equitable access to care across states for mothers with OUD and neonates with opioid withdrawal. Passing with unanimous support, this Act directs federal agencies to collect and disseminate strategies and best practices to prevent and treat maternal OUD, as well as provide recommendations for diagnosing and treating neonates with NAS. The Act also requires the Department of Health and Human Services (HHS) to address research gaps in prenatal opioid use and NAS.

As the culture of research with neonates developed, the team increased advocacy efforts by serving on multiple regional and national advisory panels addressing pediatric drug and device research (including those related to NAS) for the NIH, FDA, and SAMSHA. In 2015, the Critical Path Institute launched the International Neonatal Consortium (INC) with the team playing a leading role in establishing this public–private partnership. In 2017, the INC published guidelines for conducting pharmaceutical trials with neonates [23] and key stakeholders introduced the Promoting Life Saving New Therapies in Neonates Act, which incentivizes pharmaceutical companies to develop neonatal drugs [24].

In 2018, the team published results from its multisite clinical trial showing that neonates with NAS treated with methadone had better short-term outcomes compared to those treated with morphine, including shorter length of hospital stay and length of treatment [3]. Around the same time, Kraft et al. reported a single-site study indicating that sublingual buprenorphine for NAS was associated with shorter length of stay and shorter duration of treatment compared to oral morphine in neonates [25]. Earlier findings by researchers at another organization demonstrated that prenatal exposure to buprenorphine was associated with shorter length of treatment for NAS when compared to methadone, but buprenorphine’s limitations included a higher study dropout rate of mothers [26] and a high alcohol preservative content for neonates. In 2020, the American Academy of Pediatrics (AAP) guidelines for NAS cited the Davis et al. and Kraft et al. studies as evidence that longer-acting opioids (methadone and buprenorphine) are superior first-line pharmacologic treatments for NAS compared to morphine, although the AAP noted the concerns about the increased alcohol content [3,13,25].

Despite new guidelines for clinical treatment, expanded clinical research efforts with neonates, and legislation to incentivize the development of neonatal drugs, a commercially available formulation of either methadone or buprenorphine that is safe for neonates is still lacking. To address this, the team approached a pharmaceutical company in 2020 to develop an alcohol-free formulation of buprenorphine, which is being studied in a multicenter randomized clinical trial supported by NIH (JMD, personal communication).

## Facilitators and Barriers

### Facilitators

Four facilitators emerged which could be adopted for other research efforts (Table 1). Two facilitators are specific to NAS research and two pertain to creating the context that supports research and implementation of findings.

**Table 1.** Impact of research on NAS at one academic medical center

Category*	Indicator*	Description	Status
**Evidence-base for pharmaceutical treatment**			
Clinical and medical	Guidelines	Published guidelines recommending longer-acting opioids (e.g., methadone) as first-line agent, rather than most often used prior to treatment [13]	Demonstrated
	Therapeutic procedures	Findings influenced a shift from using morphine to using longer-acting opioids (e.g., methadone) [13]	Demonstrated
	Drugs	Ongoing testing of commercially available neonate-safer formulation of longer-acting opioid (buprenorphine)**	Potential (ongoing clinical trial expected to be completed in 2025)
Community & Public Health	Health Care Quality	Treatment with longer-acting opioids (e.g., methadone) reduced length of pharmaceutical treatment [3]	Demonstrated
Economic	Cost Savings	Treatment with longer-acting opioids (e.g., methadone) reduced length of stay, which could reduce costs of NAS treatment [3]	Potential
**Evidence-base for non-pharmacological treatments**			
Clinical and medical	Therapeutic procedures	Contributed to dissemination and implementation of the Eat, Sleep, Console (ESC) tool statewide and internationally [27]	Demonstrated
Community & Public Health	Health Care Quality	Use of the ESC tool reduced the need for pharmacological treatment of neonates with NAS [27]	Demonstrated
Economic	Cost Savings	Use of the ESC tool shortened length of stay, which could reduce costs of NAS treatment [27]	Potential
**Culture of research with neonates, for NAS and beyond**			
Policy and legislative	Expert testimony and legislation	Legislation requiring research on prenatal opioid use and NAS [22]	Demonstrated
		Legislation requiring neonatal expertise at the Food and Drug Administration (FDA) deliberations [30]	Demonstrated
		Legislation to incentivize development of neonatal pharmaceuticals [24]	Demonstrated
	Committee participation and policies	Broad stakeholder engagement of FDA’s Neonatal Advisory Committee to share knowledge and reduce inefficiencies	Demonstrated
		Broad international stakeholder engagement through Critical Path Institute’s International Neonatal Consortium to reduce inefficiencies	Demonstrated
Clinical and medical	Investigative procedures	Published guidelines for conducting clinical trials with neonates to provide a common roadmap for investigators and regulators to follow [23]	Demonstrated
		Developing first international database of real-world neonatal data to facilitate evidence base for neonatal care	Potential (anticipated to be in use by 2025)

* Categories and indicators follow the Translational Science Benefits Model (TSBM) developed by the Institute of Clinical & Translational Sciences at Washington University School of Medicine. “Demonstrated benefits are those that have been observed and are verifiable. Potential benefits are those that are logically expected with moderate to high confidence” [19].

** Source: JMD, personal communication.

First, despite the history of stigma related to opioid use and addiction, the opioid crisis in the early 2000s garnered widespread societal interest in identifying solutions. The research team attributes some of their success to “*being in the right place at the right time because then there was this explosion of the opioid epidemic in the middle of us looking at [NAS]*.” In addition, as opioid use increased among Caucasians (to a greater extent than Blacks and Hispanics) [12] and therefore “*became more pervasive in households that had the ability to have a voice about it,*” societal awareness and interest in addressing the issue increased, resulting in significantly more funding for the research. Changes in how providers, patients, and communities feel about opioid use disorder also potentially supported the feasibility of the case; one team member shared, “*…it has taken a lot of cultural shift and acceptance of this as a disorder, not only in the healthcare community but also in the local communities*.”

Second, effective interdisciplinary teams were crucial to achieving research success. Team members for the methadone-related clinical trial included investigators with expertise in neonatology, neurodevelopment, genomics, and pharmacy; site PIs and coordinators with expertise in conducting clinical trials; two statisticians for research design and data analysis; and an off-site company assisting with randomization and data management. Team members described mutual respect for each other’s areas of expertise. For example, the lead investigator credited *“a great deal [of his] success”* to the statisticians on the team, and a statistician described the lead investigator as valuing statistical input*: “And they did listen to us* … *I felt like I had something to contribute.*”

Third, creating the context to support research on NAS relied on connecting clinical care, research, and advocacy efforts. A bedside clinical question about disparate outcomes among neonates with NAS sparked research that led to advocacy to overcome societal stigma related to addiction and remove policy barriers related to conducting research with neonates. Connecting these three ‘pillars’ created “*a loop”* in which *“the advocacy part reinforces that you need to do more research”* which in turn, supports the most effective clinical care. Efforts to create this loop were supported by important mentors who acted as “*role models*” and “*really made a difference*.”

The development and testing of an alcohol-free formulation of buprenorphine for neonates provides one example of the impact of this reinforcing loop. The team’s advocacy efforts helped pass legislation requiring research on prenatal opioid use and NAS and established incentives for the creation and study of pediatric drug formulations. These incentives promoted the pharmaceutical company’s development of an alcohol-free formulation of buprenorphine for neonates. However, the company encountered barriers with engaging site investigators to conduct industry-sponsored research. To overcome this barrier, the team connected the pharmaceutical company with the NIH. With the NIH leading a trial, investigators would be more interested in participating, the company would supply the study drug free-of-cost, and the team would help guide the research.

Finally, engaging a broad range of stakeholders, including legislators, regulators, other government agencies, pharmaceutical companies, advocacy groups, and clinicians, was essential for establishing a culture to support research efforts and clinical translation of these findings. The research trajectory has been “*all about relationships… [If] you want to do great science, you must work well [together]*.” Collaborating with legislators to pass foundational laws and with regulators on clinical trial designs was key. Work with the INC included facilitating inter-stakeholder conversations between pharmaceutical companies and the FDA (in the precompetitive space and being product agnostic) so that the two parties could “*talk about outcome measures for clinical trials, how to make clinical trials work better in neonates, and obtaining relevant input from the FDA”,* thereby enabling efficient use of research resources.

As leaders within INC, the team also engaged international representatives from academic institutions, government and regulatory agencies, pharmaceutical companies, neonatal nursing organizations, and family/advocacy organizations. This multi-stakeholder Consortium collaboration has been, “*all about developing those relationships and leveraging them.”* An example of the impact of this collaboration was the FDA-funded creation of an international database of neonatal clinical trials and electronic health records to create real-world evidence and catalyze evidence-based healthcare. While the broad-based advocacy extended beyond NAS, this diverse stakeholder involvement supported a culture of research with neonates that was important for continuing NAS-related research.

Implementation of research results also required engagement of various clinician groups. For example, collaborators within the Neonatal Quality Improvement Collaborative of Massachusetts [27] undertook a statewide initiative to implement the Eat-Sleep-Console (ESC) model, focused on increasing rates of rooming-in, skin-to-skin care, and breastfeeding for infants with *in-utero* drug exposures. “*ESC started at one center in the state. And now … almost…50 percent of centers in the state have adopted [an] ESC model of care and … monitoring*.” Implementation has expanded internationally as well. “*Talking to collaborators in Australia or UK, they are starting to implement these modalities. Non-pharma care is not a new concept. …But it is kind of being looked at in a more research-oriented…manner, to say, ‘Okay, now we’ve looked at it. Let’s translate it.’”*

### Contribution of Clinical and Translational Science Award (CTSA) Hub

The local CTSA program assisted the research in several ways. Perhaps most importantly, the CTSA provided access to consistent research resources and expertise, including crucial support prior to receiving funding awards. For the clinical trial, the team’s two statisticians and its Data and Safety Monitoring Board (DSMB) were part of the CTSA’s infrastructure, specifically its Biostatistics, Epidemiology, and Research Design Center and Regulatory Knowledge and Support program. The CTSA also provided team members with education and mentorship in clinical and translational research.

### Barriers

Although the facilitators and local CTSA infrastructure supported successful translation, five significant barriers slowed the research and the efficient translation of findings into practice (Table 2). Perhaps the foremost barrier to translating research findings on pharmacological treatments for NAS into clinical care has been the lack of commercially available and alcohol-free formulations for methadone or buprenorphine for neonates [17].

**Table 2.** Barriers encountered and team strategies used

Barrier	Strategy
**Translating findings**	
Lack of commercially available, neonate-safer drug formulation of longer-acting opioid (methadone or buprenorphine), which research had found to be superior to most common drug treatment (morphine)	Advocated for legislation and policy change to incentivize and facilitate pediatric drug development [24,30] Engaged pharmaceutical company to develop a neonate-safer formulation of longer-acting opioid (buprenorphine) Facilitated collaborations between funder and pharmaceutical company to support feasibility of clinical trial for the new formulation.
**Conducting research for neonatal abstinence syndrome (NAS)**	
*Legal and regulatory barriers*	
Food and Drug Administration (FDA) requirement for new drug formulation for research despite ongoing use in clinical care	Developed an alcohol-free liquid formulation using methadone powder for research purposes The alcohol-free product needed to be tested extensively by an independent laboratory approved by FDA prior to use in neonates
Transportation and storage restrictions for regulated study drug	Obtained Drug Enforcement Administration (DEA) approval to move methadone to trial sites
Mandated reporting requirements regarding illicit / recreational drug use of research participants, which undermined trust	Advocated for increased confidentiality protection for pregnant persons participating in research regarding opioid use disorder (OUD) and NAS by publishing an article with an FDA representative[28]
*Recruitment challenges*	
Stigma and mothers’ concerns about potential negative side effects of study drug for neonate	Engaged obstetricians providing antenatal care to the mothers (a trusted source) to introduce the study and obtain informed consent. Added additional sites and expanded the inclusion criteria (e.g., including mothers who used illicit drugs during pregnancy) in order to enroll more subjects.
Controlled study design with narrow inclusion criteria (requiring mothers to be in an OUD treatment program or receiving an opioid for chronic pain treatment, but not using illicit drugs)	
*Funding environment*	
Episodic funding limited the ability to retain research staff between funding awards	Although individual research teams have limited ability to overcome this barrier, the team utilized no-cost study design and statistical guidance services supported by the local Clinical and Translational Science Award (CTSA) as a partial solution to continuity of research staff and projects

Three barriers emerged specific to research on pharmacological treatments for NAS. First, legal and regulatory barriers created delays and reduced feasibility of the research. For example, despite widespread use of the adult formulation of methadone to treat NAS in clinical practice, the FDA required a new alcohol-free formulation of the drug for use in the clinical trial. While this was a critical initial step, compounding a new formulation delayed study start-up by a year. Additionally, because methadone was considered a controlled substance, transporting and storing the study drug at trial sites was complicated, and mandatory reporting requirements meant that researchers could not guarantee confidentiality to mothers (despite a Certificate of Confidentiality being obtained). Legal jeopardy to mothers, especially in certain states where prenatal opioid exposure was considered a form of child abuse, undermined trust in research participation and ultimately the feasibility of conducting research on NAS [28].

Second, the team faced challenges in recruiting mothers to participate in the research due to stigma and fears about the investigational treatment. A team statistician shared, *“…a lot of why moms didn't want the babies randomized is because they had been on methadone and the methadone made them feel bad. And they didn't want their babies to have those same side effects*.” This fear of methadone’s side effects, together with the legal challenges associated with methadone as a controlled substance, likely contributed to the focus on buprenorphine for developing a safer neonatal formulation.

Third, as with many clinical trials, the protocol for the comparison of methadone to morphine required controlled conditions that did not align with real-world circumstances. To minimize potential confounders, the eligibility criteria required that mothers of neonates with NAS be in an addiction treatment program. However, as a team statistician described, the study was occurring as “*the opioid epidemic just exploded. And so a lot of babies who are affected by withdrawal … don't fit into this neat category of moms under treatment now*.” More generally, NAS research was slowed by the episodic nature of funding which makes it difficult to retain research staff between funding awards. One team member shared that “*looking at the existing [clinical] data … If we look at that data, we can do so much there*.” However, it has not been possible to utilize existing data due to lack of research staff outside of a specific funding award.

## Impacts

Following the Translational Science Benefits Model [19] as a guide, impacts of this case include benefits for clinical care (guidelines, drugs, therapeutic procedures, advances in investigative procedures), cost savings (social and financial cost of illness), and policy and legislative changes (expert testimony and legislation, committee participation, and policies) (Table 1).

The AAP guidelines recommending longer-acting opioids as a first-line pharmacologic treatment for NAS are evidence of one benefit emerging from this research. Ongoing clinical testing of buprenorphine is expected to improve options for pharmacologic treatment of NAS. The efforts to create safer formulations of buprenorphine are expected to expand the use of longer-acting opioids and broaden the realized outcomes of reduced length of treatment and hospital stay. These clinical benefits are also expected to significantly reduce associated healthcare costs.

Additionally, the team’s ongoing work related to non-pharmacological aspects of treating NAS supported the research and its translation by growing the team’s expertise. This work was implemented into clinical care models through statewide consortiums and international collaborator networks. These efforts were shown to reduce length of treatment and hospital stay, with their associated economic benefits [29].

Building a culture of neonatal research entailed multiple legislative and policy initiatives. These included requirements for incorporating neonatal expertise within the FDA and accelerating research on prenatal opioid use and NAS, as well as incentivizing the development of neonatal therapeutics more generally [22,24,30]. In the policy arena, INC’s broad collaboration between regulators, industry, clinicians, and families is expected to reduce barriers to the testing and approval of neonatal treatments. In addition, NAS initiatives within the HHS that have included the research team have increased the focus on protecting and improving care of the mother–infant dyad [31].

Advances in investigative procedures included INC’s best practice guidelines for conducting clinical trials with neonates, which will inform future trials on NAS. Furthermore, the development of an international database of neonatal clinical data to generate real-world evidence is expected to advance the expansion of an evidence-base for neonatal care, including and extending beyond NAS.

### Further Developments

Much work remains to improve knowledge and treatments related to NAS, particularly related to long-term effects and the potential of medical efforts to support recovery. Next steps include 1) completing trials testing a safer neonatal formulation of buprenorphine, 2) testing devices for the prevention and treatment of NAS including a low frequency vibrating mattress device and a preservative-free formulation of oxytocin with a nasal delivery device, and 3) continuing to maximize non-pharmacologic management protocols, such as the ESC tool. Ongoing facilitation of stakeholder collaborations will be vital to accelerating research translation in this area. For instance, the team now plans to align work between international regulatory agencies and industry partners so that clinical trials with neonates could be conducted in parallel across multiple countries simultaneously, greatly accelerating product approval. This would significantly enhance efficiency over the current process of conducting separate trials for each individual regulatory body. The team also has begun to support the FDA’s efforts to develop new devices for pediatric disorders by coordinating collaboration between the FDA and industry. The ultimate goal of this approach is to speed translation and reduce costs, thereby improving the sustainability of device-based research with neonates.

## Conclusion

This NAS case study elucidates key factors that contributed to successful research and translation of findings, which may be adaptable to other research contexts. This case also highlights the impact of aligning research with areas of societal interest; the power of connecting clinical care, research, and advocacy to bolster one another; and the critical roles that interdisciplinary teamwork and broad stakeholder collaboration play in overcoming obstacles to research translation.

## Supplementary material

For supplementary material accompanying this paper visit https://doi.org/10.1017/cts.2022.431. click here to view supplementary material
