# Supplementary material for: Establishing evidence-based pharmacologic treatments for neonatal abstinence syndrome: A retrospective case study
Source: J Clin Transl Sci. 2022 Jul 25;6(1):e96. doi: 10.1017/cts.2022.431 (PMC9393572; doi:10.1017/cts.2022.431)
Supplement: Supplementary file 1 [file S2059866122004319sup001.pdf]

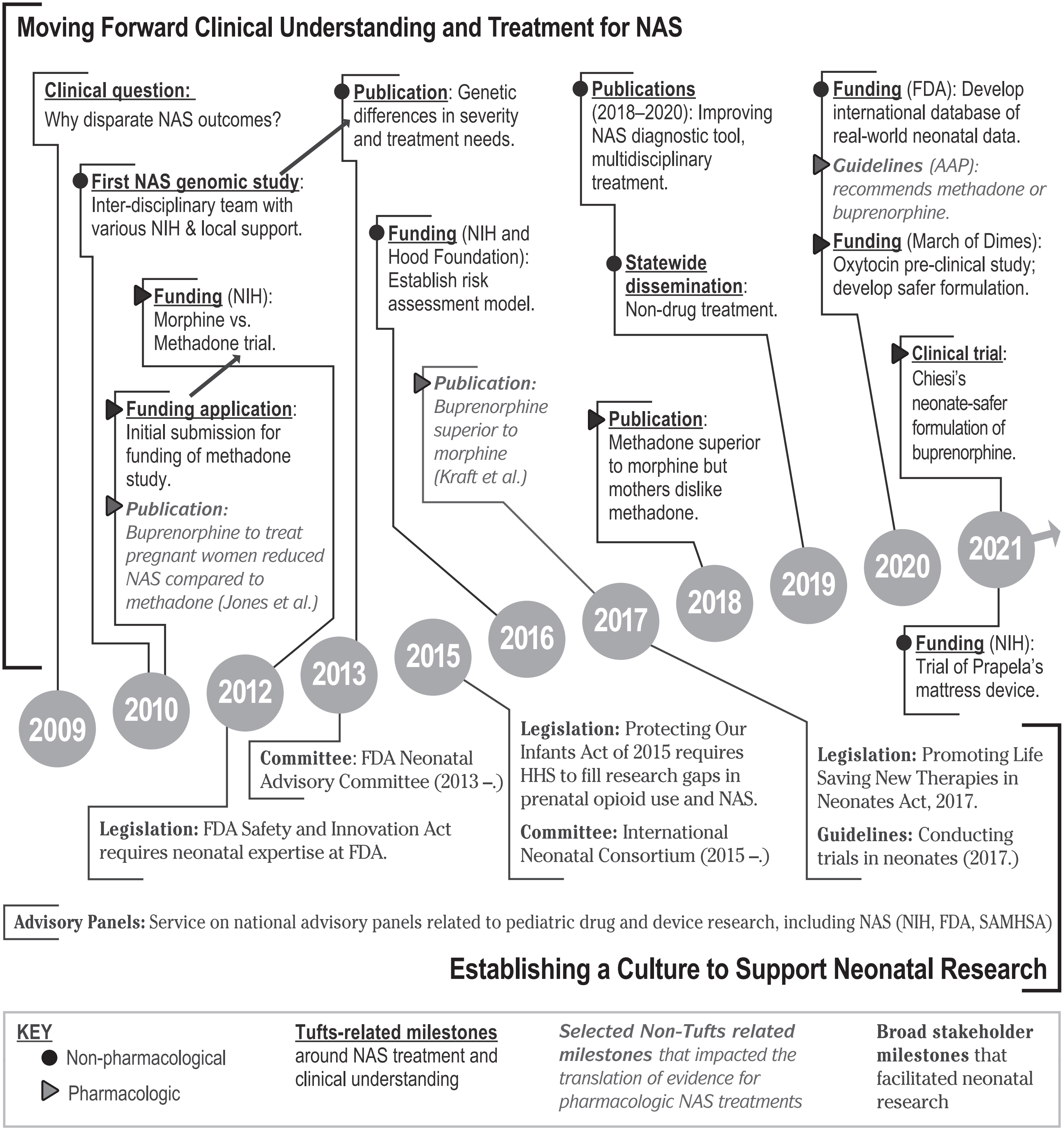
Moving Forward Clinical Understanding and Treatment for NAS
Clinical question: Why disparate NAS outcomes?
First NAS genomic study: Inter-disciplinary team with various NIH & local support.
Publication: Genetic differences in severity and treatment needs.
Funding (NIH): Morphine vs. Methadone trial.
Funding application: Initial submission for funding of methadone study.
Publication: Buprenorphine to treat pregnant women reduced NAS compared to methadone (Jones et al.)
Funding (NIH and Hood Foundation): Establish risk assessment model.
Publication: Buprenorphine superior to morphine (Kraft et al.)
Publications (2018–2020): Improving NAS diagnostic tool, multidisciplinary treatment.
Statewide dissemination: Non-drug treatment.
Publication: Methadone superior to morphine but mothers dislike methadone.
Funding (FDA): Develop international database of real-world neonatal data.
Guidelines (AAP): recommends methadone or buprenorphine.
Funding (March of Dimes): Oxytocin pre-clinical study; develop safer formulation.
Clinical trial: Chiesi's neonate-safer formulation of buprenorphine.
Funding (NIH): Trial of Prapela's mattress device.
2009
2010
2012
2013
2015
2016
2017
2018
2019
2020
2021
Committee: FDA Neonatal Advisory Committee (2013 –.)
Legislation: FDA Safety and Innovation Act requires neonatal expertise at FDA.
Legislation: Protecting Our Infants Act of 2015 requires HHS to fill research gaps in prenatal opioid use and NAS.
Committee: International Neonatal Consortium (2015 –.)
Legislation: Promoting Life Saving New Therapies in Neonates Act, 2017.
Guidelines: Conducting trials in neonates (2017.)
Advisory Panels: Service on national advisory panels related to pediatric drug and device research, including NAS (NIH, FDA, SAMHSA)
Establishing a Culture to Support Neonatal Research
KEY
Non-pharmacological
Pharmacologic
Tufts-related milestones around NAS treatment and clinical understanding
Selected Non-Tufts related milestones that impacted the translation of evidence for pharmacologic NAS treatments
Broad stakeholder milestones that facilitated neonatal research
